# Deletion of NRF2 disturbs composition, morphology, and differentiation of the murine tail epidermis in chronological aging

**DOI:** 10.1002/biof.1941

**Published:** 2023-02-11

**Authors:** Michaela Sochorová, Christopher Kremslehner, Ionela‐Mariana Nagelreiter, Francesca Ferrara, Maja Mitrovic Lisicin, Marie‐Sophie Narzt, Christina Bauer, Alexandra Stiegler, Bahar Golabi, Katerina Vávrová, Florian Gruber

**Affiliations:** ^1^ Department of Dermatology Medical University of Vienna Vienna Austria; ^2^ Faculty of Pharmacy in Hradec Králové Charles University Hradec Králové Czech Republic; ^3^ Department of Chemical, Pharmaceutical and Agricultural Sciences University of Ferrara Ferrara Italy

**Keywords:** aging, epidermis, lipid, NRF2, skin

## Abstract

NRF2 is a master regulator of the cellular protection against oxidative damage in mammals and of multiple pathways relevant in the mammalian aging process. In the epidermis of the skin NRF2 contributes additionally to the formation of an antioxidant barrier to protect from environmental insults and is involved in the differentiation process of keratinocytes. In chronological aging of skin, the capacity for antioxidant responses and the ability to restore homeostasis after damage are impaired. Surprisingly, in absence of extrinsic stressors, NRF2 deficient mice do not show any obvious skin phenotype, not even at old age. We investigated the differences in chronological epidermal aging of wild type and NRF2‐deficient mice to identify the changes in aged epidermis that may compensate for absence of this important transcriptional regulator. While both genotypes showed elevated epidermal senescence markers (increased Lysophospholipids, decreased LaminB1 expression), the aged NRF2 deficient mice displayed disturbed epidermal differentiation manifested in irregular keratin 10 and loricrin expression. The tail skin displayed less age‐related epidermal thinning and a less pronounced decline in proliferating basal epidermal cells compared to the wildtype controls. The stratum corneum lipid composition also differed, as we observed elevated production of barrier protective linoleic acid (C18:2) and reduced abundance of longer chain saturated lignoceric acid (C24:0) among the stratum corneum fatty acids in the aged NRF2‐deficient mice. Thus, despite epidermal differentiation being disturbed in aged NRF2‐deficient animals in homeostasis, adaptations in keratinocyte proliferation and barrier lipid synthesis could explain the lack of a more severe phenotype.

## Graphical abstract

NRF2‐deficient aged epidermis shows more proliferation than wild‐type controls. NRF2‐ deficient epidermis shows disturbed differentiation and the upregulation of barrier protective stratum corneum lipids, Both genotypes have elevated epidermal lysophospholipids

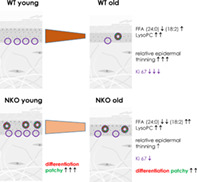

**Abbreviations**
B2M: beta‐2‐microglobulin
Cer: ceramides
Chol: cholesterol
EDC: epidermal differentiation complex
FFA: free fatty acids
FFPE: formalin‐fixed paraffin‐embedded
HPTLC: high‐performance thin layer chromatography
KC: keratinocyte
LMNB1: laminB1
LysoPC: lysophosphatidylcholines
LysoPPC: lyso‐palmitoyl‐PC
NKO: NRf2‐deficient
PPP: pentose phosphate pathway
qPCR: quantitative PCR
RMA: robust multi‐array average
WT: wild‐type

## 1 INTRODUCTION

Most of the hallmark features that describe cellular‐ and organismal aging according to the current consensus^1^ are defects that depend directly or indirectly on redox events. There is obvious redox damage associated with aging, as the accumulation of oxidized proteins,^2^ oxidative damage to DNA^3^ and specifically to telomeres.^4^ Age associated mitochondrial dysfunction and defective clearance of damaged mitochondria, due to an age related decline in auto−/mitophagic function, are further factors that enforce redox damage and are inseparable from age‐related changes in metabolism.^5^ Likewise, changes in cellular identity^6^ and the acquiring of cellular senescence—a state of arrested cell cycle that develops upon major damage and is observed more frequently in aged tissues including the skin^7^—have a significant component of redox deregulation that can be correlated to the appearance of oxidized macromolecules including proteins^8^ and lipids.^9^, ^10^

Therefore, the master regulator of cellular redox responses, Nuclear Factor (erythroid‐derived 2)‐like 2 (NRF2) is in the focus of aging research.^11^ NRF2 regulates, by controlling the transcription of its target genes, the synthesis and recycling of glutathione and of thioredoxin, which together provide redox homeostasis of proteins and other cellular molecules. Besides these direct functions NRF2 affects—directly or indirectly—other “hallmark” cellular dysfunctions that promote age‐related decline.^1^ NRF2 target genes regulate DNA damage responses (p53 binding protein 53BP1 and RAD51)^12^ and purine synthesis and thereby genomic stability.^13^ Nutrient sensing is decreased in aging due to reduced AMP‐activated protein kinase activation, which at the same time leads to decreased NRF2 phosphorylation and nuclear translocation. Conversely, metabolic responses through the pentose phosphate pathway (PPP) depend on NRF2.^13^ We have recently identified and localized activation of PPP as a response to aging promoting DNA damage in the epidermis.^14^ Furthermore other hallmark parameters including stem cell levels, intracellular communication and mitochondrial function depend on NRF2 target genes (rev. in Reference 11). The possibility to modulate NRF2 activity with drugs or through dietary interventions and natural, plant‐derived compounds created significant commercial interest also in the skin care field.^15^ In the skin, however, NRF2 has additional specific functions as it regulates keratinocyte (KC) proliferation and differentiation, it modulates epidermal specific inflammatory genes, and most importantly provides specific constitutive and adaptive cascades that are essential for the challenges the epidermis and the whole skin are exposed to.^11^, ^16^ A NRF2 gradient from basal toward the outermost differentiated strata of the epidermis provides an inside out protective barrier that at the same allows for maximal antioxidant defenses at the outside while allowing the basal cells to still undergo cell death after exposure to potentially genotoxic extrinsic stressors, especially upon short wavelength UV radiation.^17^ On the other hand, NRF2 allows the cells to react to oxidizing UV radiation and to oxidized lipids, its second messengers for the stress response.^18^ Excessive activation of NRF2, in time and extent, elicited through genetic or pharmacological intervention, caused a skin pathology characterized by a form of ichthyosis, and thickening of the stratum corneum and the epidermis, as well as an inflammatory infiltrate, and these were attributed to small proline rich proteins (Sprr2d, Sprr2h), the secretory leukocyte peptidase inhibitor (Slpi) and epigen (Epgn) upregulation.^19^ Ultimately these animals developed a chlorakne‐like phenotype with cysts in their tail epidermis. This dysregulation of selected genes of the epidermal differentiation complex (EDC) together with inflammation and dysregulation of epidermal lipids (most prominently of sebocyte‐derived triglycerides) clearly demonstrated that NRF2 activation is—especially in the epidermis—a mechanism that can have detrimental consequences when not tightly controlled. On the other hand, when components of the cornified envelope are deleted, as in the case of a loricrin knockout,^20^ NRF2 is activated and NRF2 dependent EDC members can substitute for the missing CE proteins, provided that keratin intermediate filaments were available.^21^ It was also shown that constitutive activation of NRF2 can promote a skin aging phenotype by severely dysregulating the nurnover of extracellular matrix proteins,^22^, ^23^ which together with the lack of an obvious phenotype suggests that the role of NRF2 in aging of the skin is not straightforward and needs investigation of skin aging relevant aspects that have not been yet investigated, thus we here investigated how deletion of NRF2 would affect the age related changes to murine epidermis of the tail regarding their morphology, cell proliferation, senescence markers, terminal differentiation and composition of native and oxidized lipids, termed the epi‐lipidome.

## 2 EXPERIMENTAL PROCEDURES

### 2.1 Animals

Animals were kept under standard housing conditions, with a maximum of five mice/cage, with access to food and water ad libitum, and 12‐h light and dark cycles under the institutional breeding license. They were sacrificed by cervical dislocation according to the guidelines of the Ethics Review Committee for Animal Experimentation of the Medical University of Vienna, Austria. The strain C57/BL6/J (WT) and NRF2−/− mice (NKO)^24^ on the same genetic background were used in this study. Animals were genotyped with primers (WT fwd 5'TGG ACG GGA CTA TTG AAG GCT G; WT rev 5'CGC CTT TTC AGT AGA TGG AGG; KOLacZ rev 5'GCG GAT TGA CCG TAA TGG GAT AGG) amplifying the wildtype and the targeted genomic sequeces, respectively, using the protocol suggested by the provider of the mouse strain RIKEN, Saitama, Japan.

### 2.2 Primary keratinocyte culture from mouse tail

Primary keratinocytes were isolated from the tails of young WT and NKO mice as described before.^25^ The cells were cultured with keratinocyte growth medium‐2 (KGM‐2; Lonza, Basel, Switzerland) and experiments were performed without further passaging.

### 2.3 Quantitative PCR

RNA was isolated from mouse epidermis with TriFast Reagent (VWR Peqlab) according to the manufacturer's instructions. The RNA cleanup and concentration was performed using the RNeasy MinElute Cleanup Kit (Qiagen) according to the manufactures's instructions. 400 ng of total RNA was reverse‐transcribed with the iScript cDNA Synthesis Kit (Bio‐Rad, Hercules, CA). Quantitative PCR (qPCR) was performed using the LightCycler480 and the LightCycler 480 SYBR Green I Master (Roche, Basel, Switzerland) with a standard protocol described before.^18^ Relative quantification was performed according to the model of Pfaffl et al.^26^ and the expression of the target genes was normalized to the expression of beta‐2 microglobulin.

### 2.4 Microarray mRNA expression analysis

Total RNA was extracted from mouse keratinocytes with RNeasy 96 system (Invitrogen/Life Technologies, Grand Island, NY) according to the manufacturer's instructions. The RNA cleanup was performed with the RNeasy MinElute Cleanup Kit (Qiagen) according to the manufactures's instructions. Two hundred nanograms of each sample were used for gene expression analysis with Affymetrix (Sta. Clara, CA, USA) Mouse gene 2.0 ST arrays. Hybridization and scanning were performed according to manufacturer's protocol (http://www.affymetrix.com) and robust multi‐array average (RMA) signal extraction and normalization were performed using custom chip description file. The experiment was performed on biological triplicate samples. Data are submitted to the GEO repository. Gene sets (distinct gene symbol) with a (mean) RMA value of less than 50 in all conditions were excluded as not detectable. Data were analyzed through the use of IPA (QIAGEN Inc., https://www.qiagenbioinformatics.com/products/ingenuity‐pathway‐analysis), and pathways with an *z*‐score above 2 are displayed. Significance between the groups of manually selected genes displayed was determined by ANOVA.

### 2.5 Primer sequences

lamin B1 (Lmnb1: forward: 5'‐cagattgcccagctagaagc‐3' reverse: 5'‐ctgctccagctcttccttgt ‐3'); cyclin‐dependent kinase inhibitor 1A, p21cip1 (P21: forward: 5'‐gtacttcctctgccctgctg‐3' reverse: 5'‐tctgcgcttggagtgataga‐3'); beta‐2 microglobulin (B2m forward: 5′ attcacccccactgagactg reverse: 5'‐tgctatttctttctgcgtgc‐3'); p16inkp4 (p16 forward: 5'‐cgacgggcatagcttcag‐3' p16 reverse 5′‐acgctagcatcgctagaagtg‐3′); NQO1 (nqo1 forward: 5′‐gaagctgcagacctggtgat‐3' Nqo1 reverse: 5′‐ttctggaaaggaccgttgtc‐3′).

### 2.6 Histology and immunofluorescent staining

Formalin‐fixed paraffin‐embedded (FFPE) tissue sections were cut at a thickness of 5 μm and, after antigen demasking, were incubated overnight at 4°C with the antibodies in PBS with 2% BSA and 10% preimmune serum for blocking of unspecific binding. The next day, the sections were washed and incubated with the respective secondary antibodies. Alexa Fluor® 546 goat anti‐rabbit, A11035 (diluted 1:500 in PBS with 2% BSA), for 30 min at room temperature and counterstained with 2 μg/mL Hoechst‐33258 (Molecular Probes, H1398) to visualize the nuclei and mounted in Permafluor (Thermo Scientific, TA‐030‐FM) for immunofluorescence microscopy. Images were acquired with identical settings using a BX63 upright microscope equipped with an UC90 9‐megapixel camera operated via the cellSens software at 40× magnification (all Olympus). Subsequently the epidermal staining was evaluated in at least four fields of view in sections of three different mice per genotype and age group.

### 2.7 Image analysis of histological and immunofluorescence stained FFPE sections

All image analysis was performed with ImageJ (version 1.53q). Epidermal thickness was measured at five sites per field of view with the “straight line” tool starting perpendicular from the basement membrane to the end of the granular layer. LaminB1 intensity was measured in a virtual cross section through the basal cells that was created with the “segmented line” tool. Subsequently the “Plot Profile” of the cross section was generated, the data set was transferred into Microsoft Excel. After background subtraction the mean intensity per μm was calculated from the sum of positive intensities and the length of the line segment.

### 2.8 Isolation of epidermis and stratum corneum

The skin from tail was stripped off using tweezers right after mice were sacrificed. Surface of the skin was briefly washed with acetone to remove lipophilic contaminants. The skin was cut into smaller pieces and incubated in dispase II solution for 1 h at 37°C. Afterward, epidermis was separated from dermis using tweezers. Epidermis was further processed for isolation of the stratum corneum.

Stratum corneum was obtained by trypsin treatment. Epidermis was placed on 0.25% trypsin solution with basal layer site facing down and incubated at 37°C for 3 h or overnight. Intact SC sheets were twice washed with water to remove the degraded keratinocytes. The SC sheets were dried, weighed and free SC lipids were extracted (see the following sections).

### 2.9 Isolation of SC lipids

Lipids were isolated using modified Bligh and Dyer method.^27^ The SC samples were extracted with 1 mL chloroform/methanol 2:1 (v/v) per mg of SC for 2 h, filtered using PTFE‐syringe mini filters (Millipore) and transferred to a new glass vial. The procedure was repeated with 0.5 mL chloroform/methanol 2:1 (v/v) per mg of SC for 1 h. Extracted solutions were combined and concentrated under a stream of nitrogen. The lipids were dried and stored at −20°C under argon atmosphere.

### 2.10 Analysis of SC lipids

#### 2.10.1 HPTLC of main barrier lipids

The lipid analysis was performed on silica gel 60 HPTLC plates (20 × 10 cm^2^; Merck, Darmstadt, Germany). The extracted SC lipids were dissolved in a defined amount of chloroform/methanol 2:1. Sample solutions were sprayed on the plate using a Linomat V (Camag, Muttenz, Switzerland). Standard lipids (cholesterol, lignoceric acid, ceramide EOS, NS, EOP, NP, AS, AP; CerEOS and CerEOP) were synthetized^28^ and were dissolved in chloroform/ methanol 2:1 and combined in the given concentration. To generate a calibration curve, standard lipid mixture was applied in different volumes on the plate together with analyzed samples. To separate main barrier lipids, the plate was developed 2 times to the top with chloroform/methanol/acetic acid 190:9:1.5 (v/v/v) mobile phase in a horizontal developing chamber. The lipids were visualized by dipping in a derivatization reagent (7.5% CuSO_4_, 8% H_3_PO_4_, and 10% methanol in water) for 10 s and heating at 160°C for 20 min and quantified by densitometry using TLC scanner 3 and VisionCats software (Camag, Muttenz, Switzerland). The sum of SC lipids was calculated from adding the calibrated results for FFA, Chol and Cer measurements.

GC–MS of FFA–SC extracts corresponding to 0.5 mg SC were analyzed by gas chromatography coupled to mass spectrometry as a commercial service by Synelvia SAS, (Labège, France) as in.^29^

### 2.11 Analysis of epidermal phospholipids

Isolated mouse epidermis (150 mg) was cooled on ice, purged with argon, and homogenized in Precellys (Bertin, Villeurbane, France) homogenizer tubes in 1 mL of methanol with 3% (v/v) acetic acid and 0.01% BHT at 6800 speed setting in two runs of 30 s. The supernatant obtained by centrifugation (12,000 rpm, 10 min, 4°C) was supplemented with 15 ng DNPC (Avanti Polar Lipids, Alabaster, AL, USA) as internal standard and subjected to liquid–liquid extraction with hexane for removal of neutral lipids. Relative quantification of oxidized phospholipids was performed by reversed‐phase High Performance Liquid Chromatography (HPLC) coupled to electrospray ionization MS/MS as in Reference 30. Phospholipid hydroperoxides were tentatively identified based on their precursor and product ions and co‐elution with phospholipid species that were produced by non‐enzymatic oxidation from pure synthetic phospholipids (Avanti Polar Lipids) containing specific sn‐1 saturated residue (palmitoyl or steaoryl) and sn‐2 PUFA residue (linoleoyl or arachidonoyl). Nonoxidized phospholipids and lysophospholipids were identified based on their precursor and product ions, as well as comigration with commercial standards (Avanti Polar Lipids). Relative abundance was determined by normalizing the peak areas of the investigated oxidized species to the peak areas of the di‐palmitoyl‐PC.

## 3 RESULTS

**FIGURE 1:**
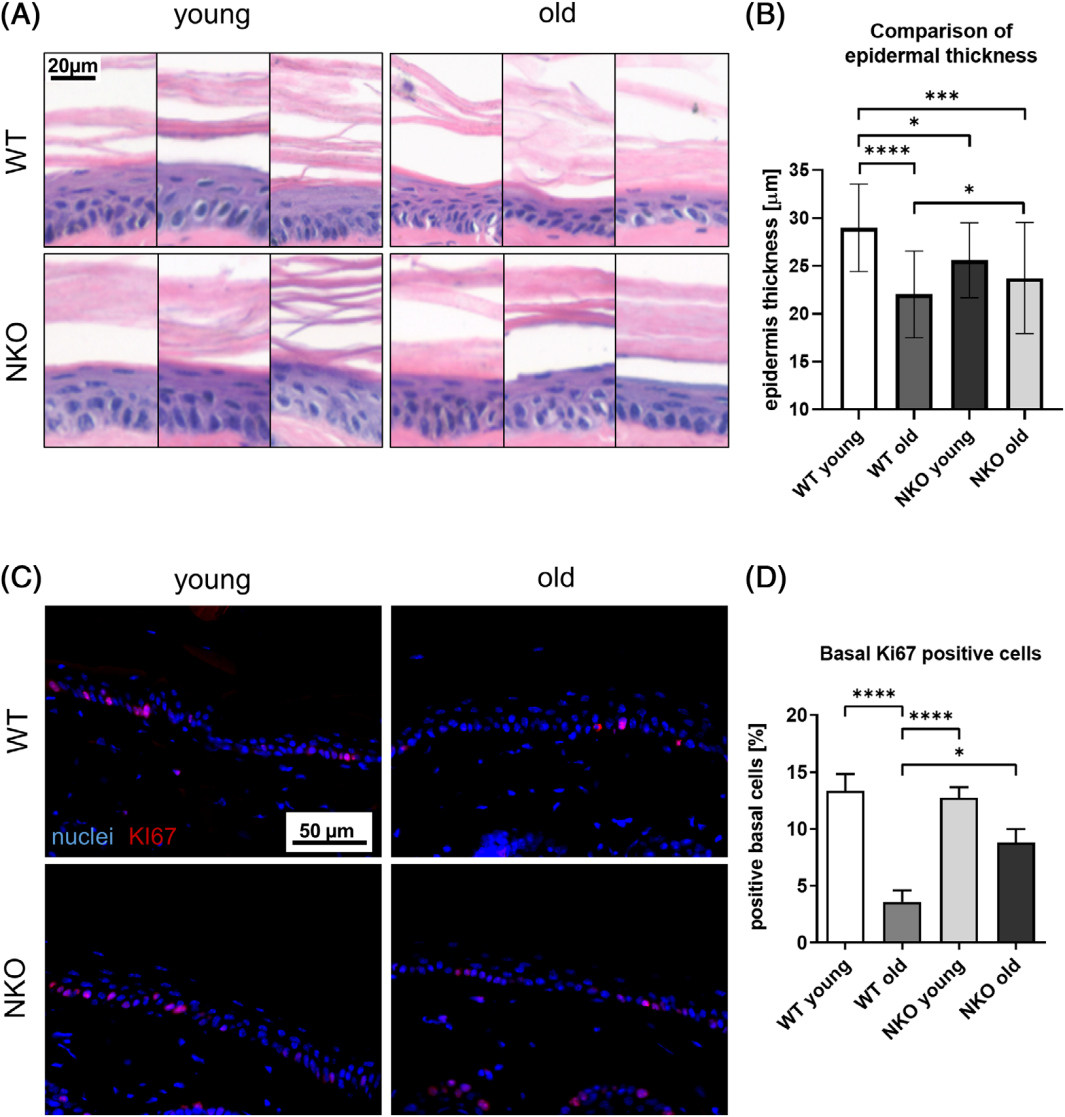
Quantification of thickness and proliferation in cross sections of murine tail epidermis. (A) Representative hematoxilin‐eosin stain micrographs of murine tail cross sections used for measurement of epidermal thickness in young and old wild‐type (WT) and Nrf2‐deficient (NKO) animals. Scale bar: 20 μm. (B) Quantification results of epidermal thickness shown as bar graph. Epidermal thickness was measured at five points in three images per mouse, *n* = 3 per genotype and age. (C) Representative images of murine tail cross sections immune fluorescently stained for the proliferation marker KI67, nuclei counterstained with Höchst reagent. Scale bar: 50 μm. (D) Quantification of percentage of KI67 positive cells in the basal layer of the epidermis. Asterisks indicate statistically significant differences (*p < 0.05, ***p < 0.001, ****p < 0.0001, one‐way ANOVA with Tukey's correction for multiple comparisons. Results are depicted as mean value with standard deviation.)

When we investigated the average thickness in cross sections of murine tail epidermis adjusted to anatomical site, we observed a significant, age‐related decrease in mean epidermal thickness when comparing young (6 ± 1 month) to old (18 ± 2 month) wild‐type animals. In NRF2‐deficient (NKO) animals epidermal thickness was lower in the young individuals, but the decrease in epidermal thickness at advanced age was not significant compared to the young NKO animals (Figure 1A,B). We then quantified the percentage of dividing (Ki67 positive) cells in the basal layer, and again found a significant age‐related decrease in the old wild‐type animals, which was less pronounced and not significant in the NRF2‐deficient samples (Figure 1C,D).

**FIGURE 2:**
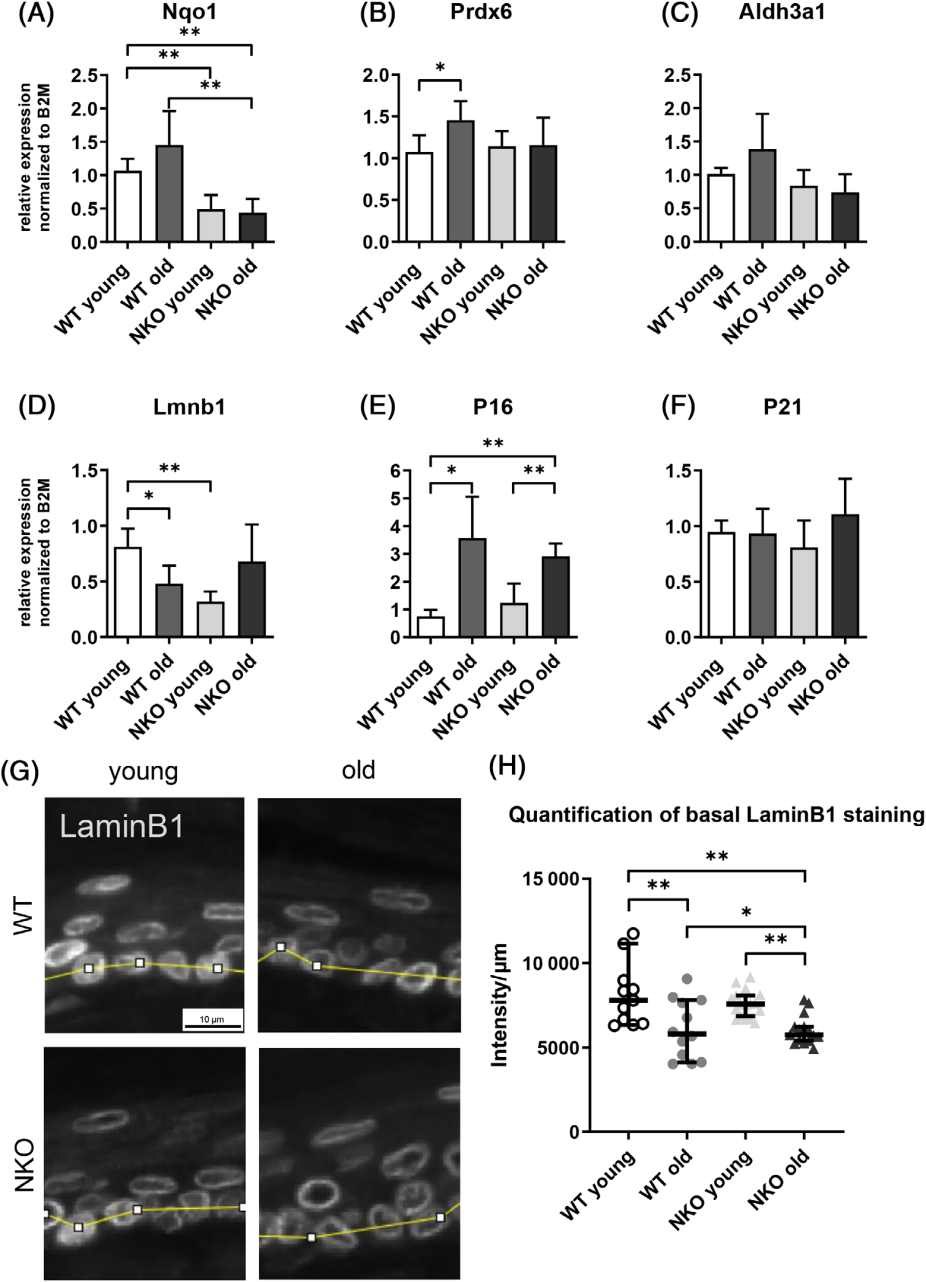
Gene expression of selected epidermal Nrf2 targets and senescence markers. Relative gene expression of the Nrf2 targets Nqo1 (A), Prdx6 (B), Aldh3a1 (C) and the senescence markers LaminB1 (D), p16 (E), p21 (F) on mRNA level in murine epidermis quantified by qPCR and normalized to the expression of beta‐2‐microglobulin (B2M). Asterisks indicate statistically significant differences (*p < 0.05, **p < 0.01, one‐way ANOVA with Newman–Keuls correction for multiple comparisons. Results are depicted as mean value with standard deviation. *N* = 4). (G) Image section of representative LaminB1 staining of mouse tail sections of young and old wildtype (WT) and Nrf2 deficient (NKO) animals. The yellow segmented line indicates the cross section through the basal layer used for LaminB1 intensity measurement. Scale bar: 10 μm. (H) Plot of background subtracted average LaminB1 staining intensity per micrometer through a cross section of the basal layer. Quantification data from 4 field of view per mouse, 3 mice per genotype & age group. Asterisks indicate statistically significant differences (*p < 0.05, **p < 0.01, one‐way ANOVA with Tukey's correction for multiple comparisons. Results are depicted as median with 95% confidence interval.)

To characterize the differences between the genotypes on the mRNA level, we investigated expression of selected epidermal NRF2 target genes (Nqo1, Prdx6, Aldh3a1), and epidermal senescence markers (LaminB1, p16, and p21) on mRNA level. The expression of the canonical NRF2 target Nqo1 was significantly reduced in the NKO in young and old epidermis (Figure 2A). Expression of Prdx 6, a NRF2‐dependent enzyme that has multiple enzymatic activities which limit phospholipid peroxidation,^31^ was elevated in old wild‐type animals, which was not the case in old NRF2 knockouts (Figure 2B). A similar trend was observed also in Aldh3a1, another epidermal NRF2 target, all supporting that NRF2 downstream functions were blunted in the knockouts and/or not induced in aged knockout epidermis (Figure 2C). In keratinocytes of the epidermis, reduced levels of the nuclear lamina protein Lamin B1 in combination with other typical markers of cell damage and cell cycle arrest are indicative of cellular senescence.^32^ We found decreased expression of Lamin B1 and an increase in p16 in aged wild‐type epidermis (Figure 2D). NRF2‐deficient epidermis showed overall reduced expression of LaminB1, which was however not decreased, but increased in the aged tissue on mRNA level, and, compared to the wild‐type samples, elevated p16 expression which was less induced by aging in NKO (Figure 2E,F).

When we aimed to corroborate the expression data on protein level, we confirmed that specific perinuclear Lamin B1 staining was decreased in the basal epidermal cells of the aged as compared to young mice. The NKO mice, however, displayed intensities similar to the respective wild‐type cohorts, with an age‐dependent decrease in LaminB1 (Figures 2G,H and S1).

**FIGURE 3:**
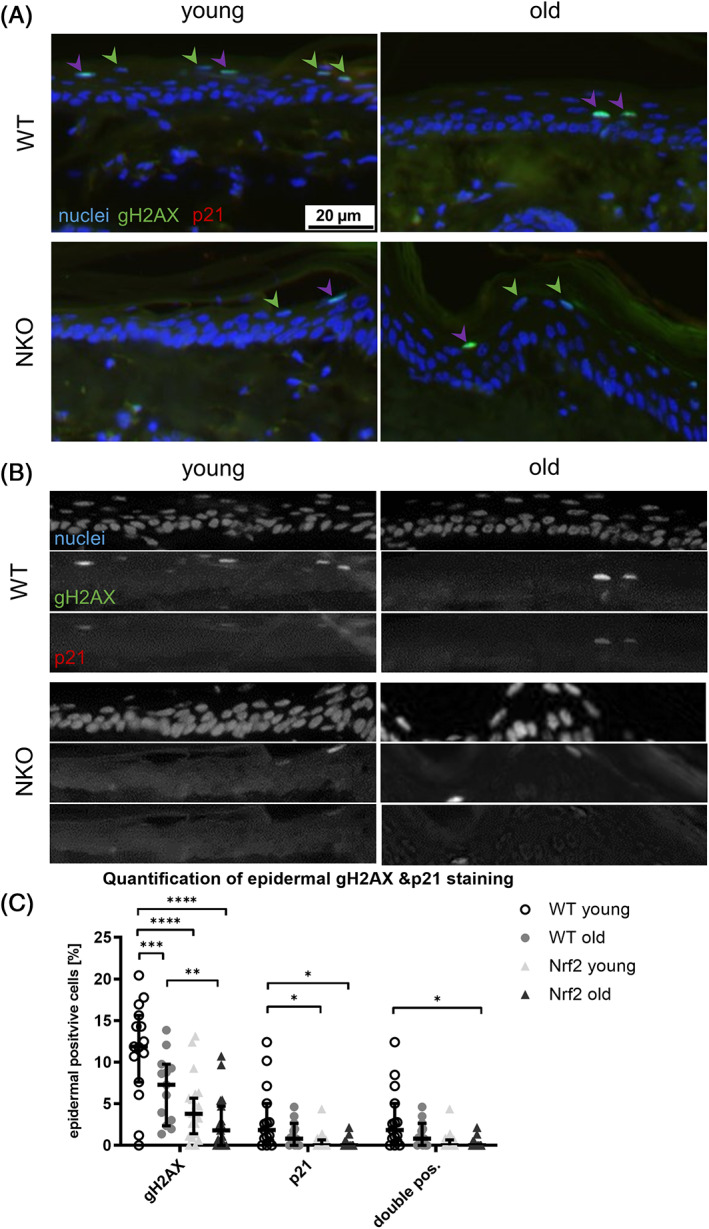
Assessment of activated DNA damage repair in epidermal cells positive for senescence marker p21. (A) Representative images of murine tail cross sections of young and old wildtype (WT) and Nrf2 deficient (NKO) animals. Shown are immunofluorescence stainings of the activated DNA damage repair marker gamma H2AX (gH2AX, green), the senescence marker p21 (red) and nuclei counterstain with Höchst reagent. Green arrow heads indicate gH2AX single positive and purple arrow heads double positive cells respectively. Scale bar: 20 μm. (B) Matrix of single channel greyscale image sections of the merged micrographs shown in A. (C) Plot of gH2AX and/ or p21 positive cells in the epidermis shown as percentage of total epidermal cells. Symbols represent quantification data from 4 field of view per mouse, 3 mice per genotype & age group. Asterisks indicate statistically significant differences (*p < 0.05, **p < 0.01, ***p < 0.001, ****p < 0.0001, one‐way ANOVA with Tukey's correction for multiple comparisons. Results are depicted as median with 95% confidence interval.)

We next assessed the numbers of epidermal cells that showed DNA damage, or rather activated DNA damage repair (DDR) and thus being positive for nuclear gamma H2AX, cells positive for nuclear p21, and double positive cells (Figure 3A–C). We did not only assess basal cells, but also suprabasal ones and those in transition to terminal differentiation but still positive for nuclear dye (mostly corresponding to the granular layer). Here we found overall a slight age related reduction of cells that were positive for DNA damage repair (gammaH2AX positive), for nuclear p21, or double positive in the WT. In the NKO, there were significantly less cells positive for gamma H2AX already in the young animals, and very few double positive cells (Figure 3C). Most of the counts were observed in the last nucleated differentiated strata rather than in the basal cells (Figure S2).

**FIGURE 4:**
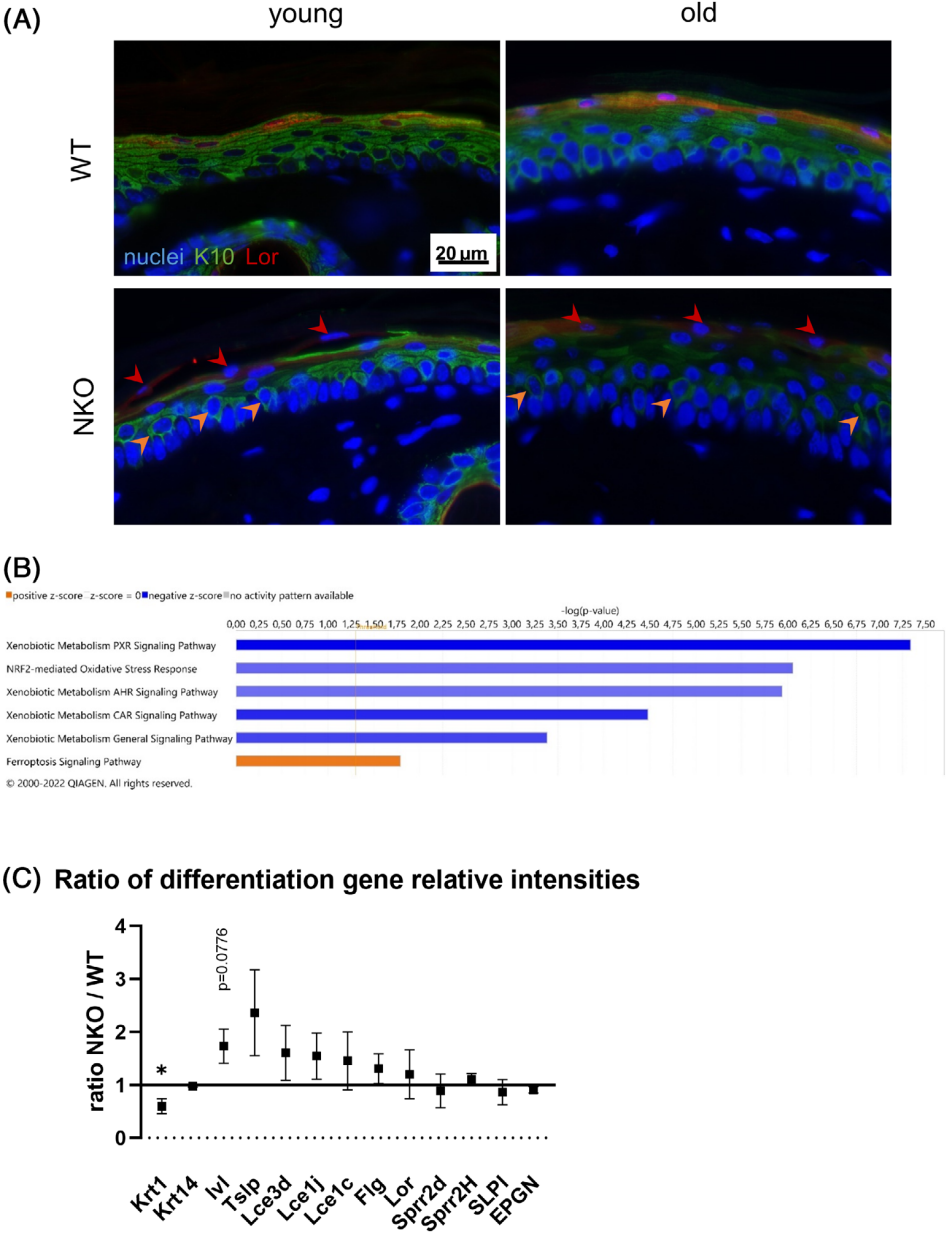
Expression of keratinocyte differentiation markers in murine tail skin and cultured cells. (A) Representative images of murine tail cross sections of young and old wild‐type (WT) and Nrf2‐deficient (NKO) animals. Shown are immunofluorescence stainings of early and late keratinocyte differentiation markers Keratin 10 (K10, green) and Loricirn (Lor, red) respectively with Höchst reagent counterstained nuclei. Red arrow heads indicate K10 negative/Lor positive cells in the granular layer while orange arrow heads highlight suprabasal keratinocytes with prolongations toward the basal layer. Scale bar: 20 μm (B) Pathways with significant *z* scores in a gene ontology analysis of cultured keratinocytes form WT and NKO epidermis. Nrf2‐ and xenobiotic pathways were down‐regulated while the Ferroptosis pathway was up‐regulated. (C) Ratio of relative intensities of differentiation related genes in transcriptomic analysis data of cultured keratinocytes from WT and NKO murine epidermis. Asterisks indicate statistically significant differences (*p < 0.05, Student's *t*‐test. Results are depicted as mean with standard error of the mean.)

As at the same time markers for proliferation were increased in the knockouts, Lamin B1 protein degradation as a marker for cellular aging was increased, but the presence of DNA damaged cells was decreased in NRF2‐deficient cells, especially upon aging, we assumed that the turnover of cells could be disturbed in the knockouts. Since NRF2 activation was recently implicated in compensating for lacking barrier proteins by inducing alternative components of the cornified envelope,^20^, ^33^ we investigated whether the expression or distribution of differentiation markers loricrin and keratin 10 would give a hint on disturbed epidermal differentiation. We found that Keratin 10 expression was much more unevenly distributed within the NKO tail epidermis samples, and that the co‐expression with loricrin in the last granular layer appeared to be uncoordinated, with more frequent appearance of loricrin positive and K10 negative cells than in the corresponding WT samples (red arrow heads, Figure 4A) and more suprabasal K10 keratinocytes with spiky protrusions toward the basal layer (orange arrow heads; Figure 4A, classification in Figure S3). We performed a transcriptomic analysis of wild‐type and NKO cultured keratinocytes to investigate whether there would be an indication on disturbed differentiation gene expression already in monolayer cells. The pathway gene ontological analysis (performed with IPA software) yielded significant *z* scores for the expected down‐regulation of NRF2‐ and xenobiotic pathways, and a positive regulation of the Ferroptosis pathway (Figure 4B). Differentiation pathways were not automatically detected by this gene ontology study, so we selected manually epidermal differentiation genes that had been implicated as being affected by NRF2 earlier in this work and in previous works by others.^19^, ^21^ Among those genes, Krt10 was significantly downregulated, whereas the detected terminal differentiation genes were upregulated by trend, suggesting that indeed differentiation regulation was disturbed in the NKO (Figure 4C). Using this dataset we also performed an analysis of genes being localized in mitochondria or affecting mitochondrial function (indicated by their GO Term “cellular component” contains “mitochondrion”) which identified reduced expression of genes required for the detoxification of reactive aldehydes and electrophiles (including mitochondrial aldehyde dehydrogenase 2 [ALDH2], a sulfide quinone reductase [Sqrdl], microsomal glutathione S transferase [Mgst1] and prominently, while not specifically mitochondrial, also Catalase [Cat], Figure S4). Of interest, also Caspase 1, a central regulator of cell death and cytokine maturation, was donwregulated in the NKO. As aging and senescence are frequently correlated with a low‐grade inflammatory phenotype^1^ we investigated in this dataset also whether lack of NRF2 would already be sufficient to affect keratinocyte cyto/chemokine expression. While we did not observe a general trend to increased cyto/chemokine gene expression, there was in interesting shift in members of the interleukin 1 family, with IL‐33 being downregulated and IL‐1a expression being increased (Figure S5). This might be of interest in further studies, as IL‐33 is known to suppress filaggrin expression^34^ and thus could contribute to dysregulation of the barrier components.

**FIGURE 5:**
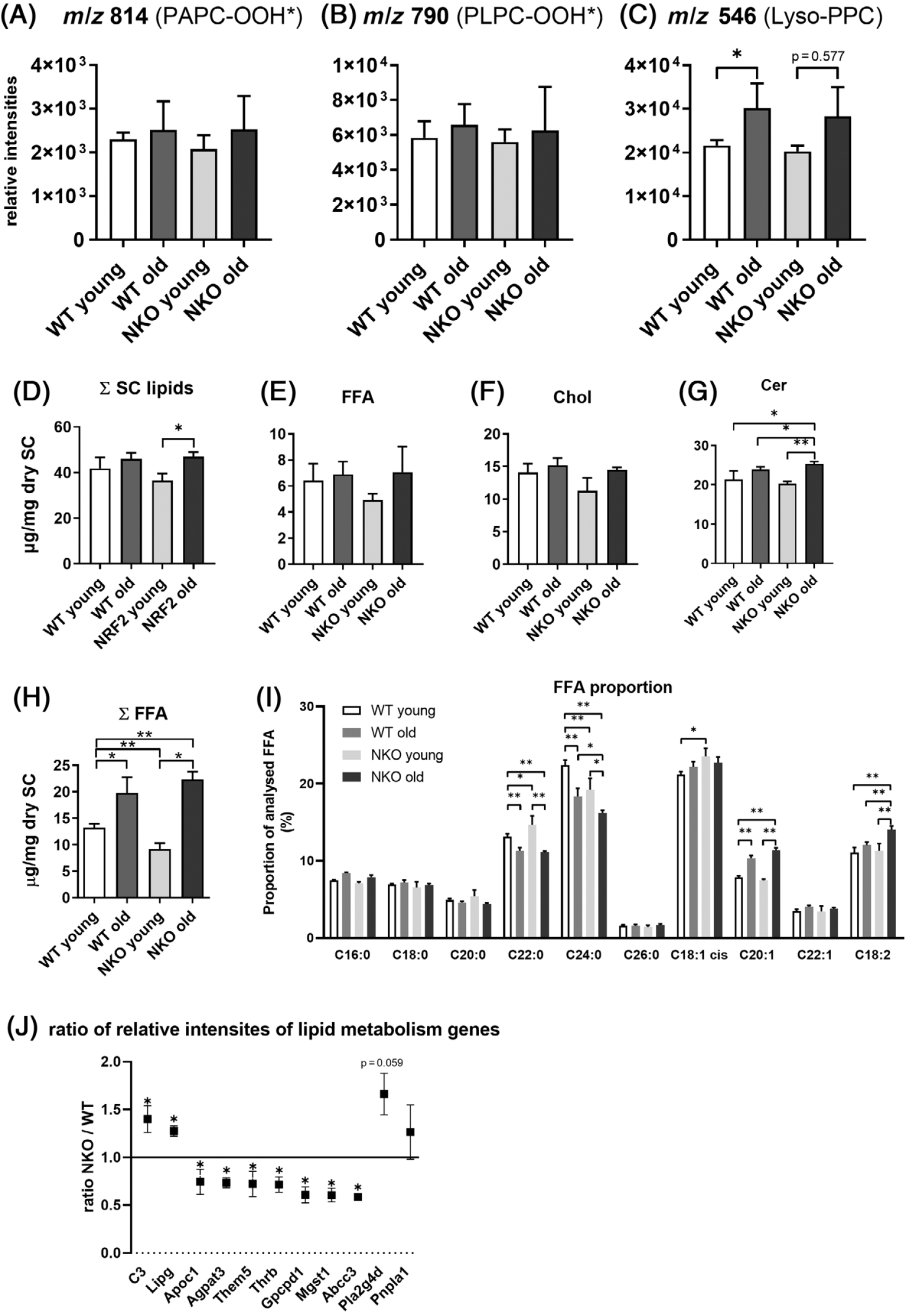
Analysis of the (Epi‐) Lipidome of Nrf2 deficient murine epidermis. Bar graphs of HPLC‐MS/MS‐based redox lipidomic quantification of the phospholipid oxidation products m/z 814 (PAPC‐OOH*) (A), *m*/*z* 790 (PLPC‐OOH*) (B) and *m*/*z* 546 (Lyso‐PPC) (C) as an indication of phospholipid peroxidation within the epidermis of young and old wildtype (WT) and Nrf2 deficient (NKO) animals. The effects of Nrf2 deficiency on the abundance of the major classes of stratum corneum (SC) lipids (D) consisting of free fatty acids (FFA, E), cholesterols (Chol, F) and ceramides (Cer, G) was analyzed by high performance thinlayer chromatography. GC–MS analysis: Calculated sum of detected FFA (H) and proportional distribution of detected FFA (I). Asterisks indicate statistically significant differences (*p < 0.05, **p < 0.01, two‐way ANOVA with Tukey's correction for multiple comparisons. Results are depicted as mean with standard deviation). (J) Ratios of relative intensities of genes with lipid metabolism relevant gene ontology terms in transcriptomic analysis data of cultured keratinocytes from WT and NKO murine epidermis. Asterisks indicate statistically significant differences (*p < 0.05, Student's *t*‐test. Results are depicted as mean with standard error of the mean.)

Next we performed analysis of phospholipids of the living layers of the epidermis and the epidermal barrier lipids, to investigate whether the disturbed differentiation was reflected in the epidermal lipid composition. We investigated whether there would be an elevated level of phospholipid peroxidation in the living layers of the epidermis, which would be expected in a tissue undergoing increased redox stress due to lacking antioxidant defenses, but also upon increased Ferroptosis. We applied a HPLC‐MS/MS redoxlipidomic method^30^ to quantify the oxidation products of the major phosphatidylcholines bearing unsaturated fatty acid chains, which are susceptible to ROS mediated or enzymatic oxidation. There was a trend toward elevated levels of PL hydroperoxides in aged epidermis, however we found comparable results in the NRF2 deficient tissues (Figure 5A,B). We also investigated whether lysophospholipids would be elevated in the aged epidermis, because we have earlier identified Lysophosphatidylcholines (LysoPC) as major lipid species elevated in senescent cutaneous cells.^10^ Indeed, Lyso‐palmitoyl‐PC (LysoPPC) was elevated significantly in aged epidermis as compared to young epidermis in the WT animals, a phenotype that was, to our knowledge not reported before. This age‐dependent increase in LysoPPC was not significant in the NRF2‐deficient tissues, however the similar trend did not suggest major differences in LysoPPC formation depending on NRF2 (Figure 5C).

Next, we investigated how selected lipids and lipid classes of the epidermal barrier were affected by the NRF2 deficiency in aging. A high‐performance thin layer chromatography (HPTLC) analysis of stratum corneum lipids, demonstrated a trend to age related increase in free fatty acids (FFA), cholesterol (Chol), and Ceramides (Cer) (Figure 5C), and a higher total lipid content of the stratum corneum, and this increase was significant and more obvious in NKO (Figure 5D–G). We then investigated with GC–MS, the regulation of the most prominent SC FFA species by age and genotype. Here we found that especially FFA 18:2 (typically linoleic acid) and FFA 20:1 (in murine epidermis typically eicosenoic acid) were elevated in the aged NKO SC, while longer chain saturated FFA 24:0 (lignoceric acid) (Figure 5I) was decreased. Linoleic acid is long known as a major factor for epidermal barrier restoration, direct precursor for acylceramides and corneocyte lipid envelope, and a precursor to arachidonic acid and thus to downstream eicosanoid synthesis.^35^

SC FFA derive mainly from hydrolysis of phospholipids but the epidermis is also inducing de novo synthesis for the restoration of the barrier.^36^ A search for lipid related genes (according to GO term) in the cultured WT and NKO KC yielded nine genes that were expressed at physiologically relevant levels and showed significant regulation (Figure 5J). Of these, one lipase (LipG) was induced, and lipase inhibitor ApoC1 was downregulated, as well as the phospholipid synthase Agpat3. Other prominent epidermal lipases were induced by trend (Pnpla1, Pla2g4d). LipG was reported recently by Cadenas et al.^37^ to be induced by cells undergoing oxidative stress for an emergency supplementation with fatty acids. The transcriptomic data did not allow to determine the mechanistic cause for the shift toward unsaturated fatty acids in the aged NKO epidermis/SC. The age related increase in epidermal lysophospholipids however is compatible with the observed increase in free fatty acids in the more differentiated strata, and the (phospho) lipases increased in the NKO keratinocytes support that this effect was elevated in aged NKO SC.

## 4 DISCUSSION

The classical study on epidermal barrier lipids in aging of human and murine epidermis by Ghadially et al.^38^ had described a decrease in total SC lipid content by about 30%, coinciding with decreased secretion of lamellar body contents and as a result decreased barrier recovery after disturbance. Therefore, the overall increase in SC lipid content we found in mice of a similar age range (18 ± 2 months) was surprising. The body site where Ghadially et al. had collected the lipids for HPTLC analyses is not specified but most likely was the sites at the flank/back where they had performed barrier functionality experiments. We had chosen tail skin, as we wanted to set our findings into relation with the earlier studies on NRF2 dysregulation, and it is thus likely that the age related changes in lipid content have a different dynamic in this body site. Another explanation would be that the Ghadially study was conducted in CrL:SKHI (hr/hr) BR hairless mice, whereas our study was done in C57/Bl6 mice. Of special interest beyond the higher amplitude of the overall lipid content increase in the NRF2 knockout mice (starting from lower levels in young individuals and ending at a higher content in the old) are the qualitative changes we observed. The content (within the analyzed FA) in the unsaturated FFA 18:2 (linoleic acid) was significantly elevated in the knockouts compared to young and to same age wild‐type animals. Linoleic acid is not only of importance as a precursor for the synthesis of arachidonic acid and thus for eicosanoid production, but has an important role in the epidermal barrier. LA has been identified long ago as a barrier lipid component in both free acylceramides and corneocyte lipid envelope and elevating the levels of LA can restore barrier defects independent of arachidonic acid synthesis.^35^

The transcriptomic data of the isolated keratinocytes in culture indicate that even in the undifferentiated, proliferating NRF2‐deficient cells there is increased (phospho‐)lipase expression. Lipase G (LipG) is an enzyme that upon oxidative stress releases FFA from PC^37^ and the by‐trend elevated phospholipases PLA2G4D and PNPLA1 have been reported to be dysregulated or mutated when differentiation or barrier function are disturbed^39^ and PNPLA1 has a special role as it acts as a transacylase which transfers linoleic acid from triglyceride to the ω‐hydroxy fatty acid in ceramides thereby generating ω‐O‐acylceramide and being essential for barrier formation and KC differentiation.^40^ The lipid metabolic enzymes downregulated in the NRF2 knockouts comprise Agpat3, an acyltransferase for epidermal phospholipid synthesis,^41^ the solute carrier ABCC3 with epidermal activity^42^ and the lipid transporter apolipoprotein C1, which causes barrier defects when overexpressed in mice.^43^ Together, the gene expression pattern of NKO KC in culture is thus compatible with increased FFA production as observed in the SC lipidome, if retained by the KC throughout the differentiation process, however we did not study expression throughout differentiation stages here. We did unfortunately not yet find mechanistic clues for the shift from long chain unsaturated FFA toward 18:2 and 20:1 in the old NRF2 deficient SC, which we assume to have a barrier reinforcing effect.

Another unexpected finding was made regarding the oxidation state of polyunsaturated phospholipids isolated from the epidermis. We had assumed that the loss of NRF2 and therefore loss of well‐established downstream mechanisms that prevent skin (phospho‐)lipid peroxidation like for example Prdx6 induction,^44^ Glutathione synthesis, regulation of Glutathione peroxidases^45^ lead to increased lipid peroxidation in the knockouts, especially in the aged tissue. We were even more expectant to observe peroxidation as the transcriptomic analysis had yielded a positive prediction for Ferroptosis and Ferroptosis appears to be affected on multiple levels by NRF2 and its downstream mediators.^46^ Another reason to expect elevated lipid peroxidation was the observed downregulation of mitochondrial genes important for aldehyde detoxification and glutathione recycling in the NKO KC. When analyzing expression of selected genes in epidermal mRNA samples we observed that lack of NRF2 reduced already the epidermal baseline mRNA expression of NQO1, whereas for PRDX6 and ALDH3 (by trend) only the age related elevation of expression was blunted in the NKO. This underlines that NQO1 is one of the target genes that are activated constantly within the NRF2 differentiation dependent antioxidant gradient,^17^ whereas activation of the other two targets would require and additional stimulus as described for PRDX6.^47^ Despite the lack of these baseline or inducible genes that counteract lipid peroxidation, the observed very weak trend for increased age related epidermal phospholipid‐hydroperoxide levels did not markedly differ between the genotypes. Whether this lack in PC‐OOH speaks against an increase in Ferroptosis cannot be stated with certainty. A redoxlipidomic profile of different types of cell death detected PE, PI and PS but not PC hydroperoxides to be elevated^48^ but other studies have shown clear involvement also of PC‐OOH in the ferroptotic process.^49^

When moving to the ultimate product of phospholipid peroxidation, we have reported recently that in fibroblasts cellular senescence leads to a massive accumulation of LysoPC.^10^ We have here, to our knowledge for the first time reported a significant aging associated increase of LysoPC in aged murine epidermis in vivo, but there was likewise no further accumulation of LysoPC in the aged NKO that would support that either senescent cells with high LysoPC content or LysoPC as the end product of lipid peroxidation would be further elevated by lack of NRF2.

The presence of senescent cells in the epidermis is not a straightforward parameter to investigate, because the nature of this stratifying epithelium with a high cellular throughput is that most cells have a limited duration of residence, and evidence accumulates that senescent keratinocytes are preferentially shed via depletion of anchoring proteins^50^ and accelerated differentiation.^51^ The nuclear lamina protein LaminB1 (LMNB1) was identified as a robust marker of cutaneous cell senescence, and a decrease in its intensity can be observed in chronological skin aging^52^ and upon induction of damage that promotes cellular senescence.^53^ Also in the normal KC differentiation process LaminB1 gets eventually degraded, thus we focused on the basal KC, and found age related decline in the characteristic perinuclear immunostaining intensity, but that was comparable in both genotypes. Defining a cell as senescent should be based on a combination of markers,^54^ and our investigation of cells that were positive for DNA damage repair (gammaH2AX positive), for nuclear p21, or double positive, suggested that there were less cells positive for gamma H2AX and almost no double positive cells in the knockouts, but it has to be noted that most of the counts were observed in the last nucleated differentiated strata rather than in the basal layer.

Taken together the data so far suggested that while the basal cells show a decrease in LAMINB1 and Lyso‐PC and thus signs of aging at the cellular level in both genotypes, the presence of DNA damage (repair) and enforced cell cycle arrest is reduced in the (thicker) epidermis of the knockouts. From that it would be thinkable that either the NRF2 deficiency protects from DNA damage, or prevents H2aX phosphorylation, or that rather the dynamics of cell removal and replenishment are different in the knockouts.

The latter more likely scenario is supported by the finding that the early differentiation marker keratin 10 displayed an irregular intensity distribution in NKO of both ages and that along the border to the stratum corneum the regular costaining with loricrin was more frequently lost in NKO than in WT. Apart from the earlier studies which identified specific EDC genes as NRF2 targets and therefore made an effect of the knockout on differentiation feasible, the transcriptomic analysis of cultured KC from both genotypes suggested an intrinsic consequence of NRF2 deficiency on differentiation, as a significant decrease in KRT10 expression was observed but also a strong while non‐significant trend for increased expression of several later stage differentiation genes. The epidermal barrier has surprising functional redundancy that can compensate for loss of single protein components including loricrin or involucrin,^54^ and the NKO do not show any sign of flakyness, itching, or increased susceptibility to skin infections as would be expected with a barrier defect. We could not directly investigate barrier function in this study, but perhaps compensatory production of barrier supporting lipids as observed here, is a more common phenomenon that also should be studied in other epidermal model systems, and it would be of importance to investigate whether these findings are observed also in human tissue models where NRF2 can be targeted.

Differentiating keratinocytes invariably end up as dead corneocytes with degraded DNA without ever dividing again and therefore there is little selection pressure for having functional DDR, and indeed many classical studies have shown less functional ability to repair DNA damage in differentiating KC.^55^ The phosphorylation of the histone H2aX which then directs the DDR machinery to sites of DNA damage can however be activated upon genotoxic stress in suprabasal differentiated cells up to at least the granular layer.^56^ We have recently observed rapid H2AX phosphorylation upon UVB irradiation throughout the living epidermis and found it spatially correlated with metabolic adaptations (pentose phosphate pathway activation) that provide reducing equivalents and nucleotide precursors.^14^ Some key proteins that regulate cell cycle arrest during DNA damage repair have however additional functions that could be also important in the context of sustaining ordered terminal differentiation.^57^ Cyclin D1, which is inhibited by p16, is localized in a differentiation‐dependent way in the cytoplasm of epidermal keratinocytes and regulates their adhesion/detachment properties^58^ but it is unknown whether p16, which accumulates in the nuclei of DNA damaged but also in senescent cells, can also negatively regulate this cytoplasmic function of Cyclin D1. It will therefore be interesting to investigate whether the observed differences in DNA damage markers and cell cycle regulating proteins contribute to the irregular differentiation phenotype.

In conclusion, the loss NRF2 as the central regulator of antioxidant responses did not cause or aggravate typical age related oxidative damage or senescence in the tail epidermis. NRF2 deficiency rather caused irregular differentiation of keratinocytes without impairing formation of the stratum corneum and without causing any obvious signs of barrier defects. Our data suggest that adaptations in cell proliferation and an adjustment of the amount and composition of the epidermal barrier lipids, especially increased presence of linoleic acid in the SC allow upholding epidermal homeostasis in aging in the absence of NRF2.

## AUTHOR CONTRIBUTIONS

*Conceptualization*: Florian Gruber and Michaela Sochorová; *Methodology*: Marie‐Sophie Narzt, Bahar Golabi, Maja Mitrovic Lisicin, Ionela‐Mariana Nagelreiter; *Software*: Christopher Kremslehner; *Validation*: Florian Gruber, Michaela Sochorová; *Formal analysis*: Michaela Sochorová, Christopher Kremslehner, Alexandra Stiegler.; *Investigation*: Francesca Ferrara Christopher Kremslehner, Christina Bauer, Maja Mitrovic Lisicin, Ionela‐Mariana Nagelreiter; resources, Florian Gruber; *Data curation*: Alexandra Stiegler; *Writing—original draft preparation*: Florian Gruber, Michaela Sochorová, Christopher Kremslehner; *Writing—review and editing*: Florian Gruber, Katerina Vávrová, Michaela Sochorová, Christopher Kremslehner; *visualization*: Christopher Kremslehner, Bahar Golabi, Christina Bauer; *Supervision*: Michaela Sochorová, Florian Gruber; *Project administration*: Ionela‐Mariana Nagelreiter, Michaela Sochorová; *Funding acquisition*: Florian Gruber, Katerina Vávrová.

## Supporting information


**Fig S1.** Comparison of LaminB1 immunofluorescence staining in the epidermis of mouse tail sections (A) Representative micrographs of mouse FFPE tail sections stained for LaminB1 taken at 20x magnification. The white dashed boxes indicate the areas used for staining quantification. Scale bar: 50 μm (B) LaminB1 staining intensity was quantified along the yellow line demarking a cross section of the basal layer. (C) Plot of LaminB1 staining intensity for the given yellow line (B). Red horizontal line indicated the background noise that was subtracted for calculation of the average LaminB1 staining intensity per μm of basal layer. Quantification data of 4 field of view per mouse, 3 mice per genotype & age group.

**Fig S2.** Percentages of gamma H2AX and p21 positive cells in the different layers of the epidermis. Relative ratios of gamma H2AX (gH2AX), p21 and double positive (++) cells in the basal (A), suprabasal (B) and terminally differentiating cells (C) within the respective layer. Symbols represent quantification data from 4 field of view per mouse, 3 mice per genotype & age group. Asterisks indicate statistically significant differences (*p < 0.05, **p < 0.01, ***p < 0.001, ****p < 0.0001, one‐way ANOVA with Tukey's correction for multiple comparisons. Results are depicted as median with 95% confidence interval.)

**Fig S3.** Classification of Keratin 10 and Loricrin staining pattern in the epidermis of Nrf2 deficient murine tail cross sections of young and old wildtype (WT) and Nrf2 deficient (NKO) animals. (A) Classification of the overlap of Keratin 10 (K10) and Loricrin (Lor) immunofluorescence staining. (B) Classification of the lack of K10 from the terminally differentiating cells in murine epidermis. (C) Evaluation of the patchiness of K10 staining in the epidermis of WT and NKO mice. Classification scheme: none (not present), moderate (single cells per field of view matching the criteria), high (a large proportion of the cells per field of view match the criteria). Symbols represent the average classification score of 4 field of view per mouse, 3 mice per genotype & age group as assessed by four independent investigators. Asterisks indicate statistically significant differences (*p < 0.05, **p < 0.01, ***p < 0.001, ****p < 0.0001, one‐way ANOVA with Tukey's correction for multiple comparisons. Results are depicted as median with 95% confidence interval.)

**Fig S4.** Transcriptomic analysis of mitochondrial genes in cultured WT and NKO keratinocytes. Ratios of relative expression levels of genes tagged with the gene ontology term (cellular component: mitochondrion) from cultured WT and NKO keratinocytes. Asterisks indicate statistically significant differences (*p < 0.05, **p < 0.01, Student's t‐test. Results are depicted as mean with standard error of the mean.)
**Figure S5.** Transcriptomic analysis of mitochondrial genes in cultured WT and NKO keratinocytes. Ratios of relative expression levels of genes tagged with the gene ontology term (molecular function ‐ chemokine OR cytokine) from cultured WT and NKO keratinocytes. Asterisks indicate statistically significant differences (*p < 0.05, **p < 0.01, Student's t‐test. Results are depicted as mean with standard error of the mean.)

## Data Availability

The data that support the findings of this study are available from the corresponding author upon reasonable request.
