# Supplementary figures and images for: Deletion of NRF2 disturbs composition, morphology, and differentiation of the murine tail epidermis in chronological aging
Source: Biofactors. 2023 Feb 11;49(3):684–98. doi: 10.1002/biof.1941 (PMC10946746; doi:10.1002/biof.1941)

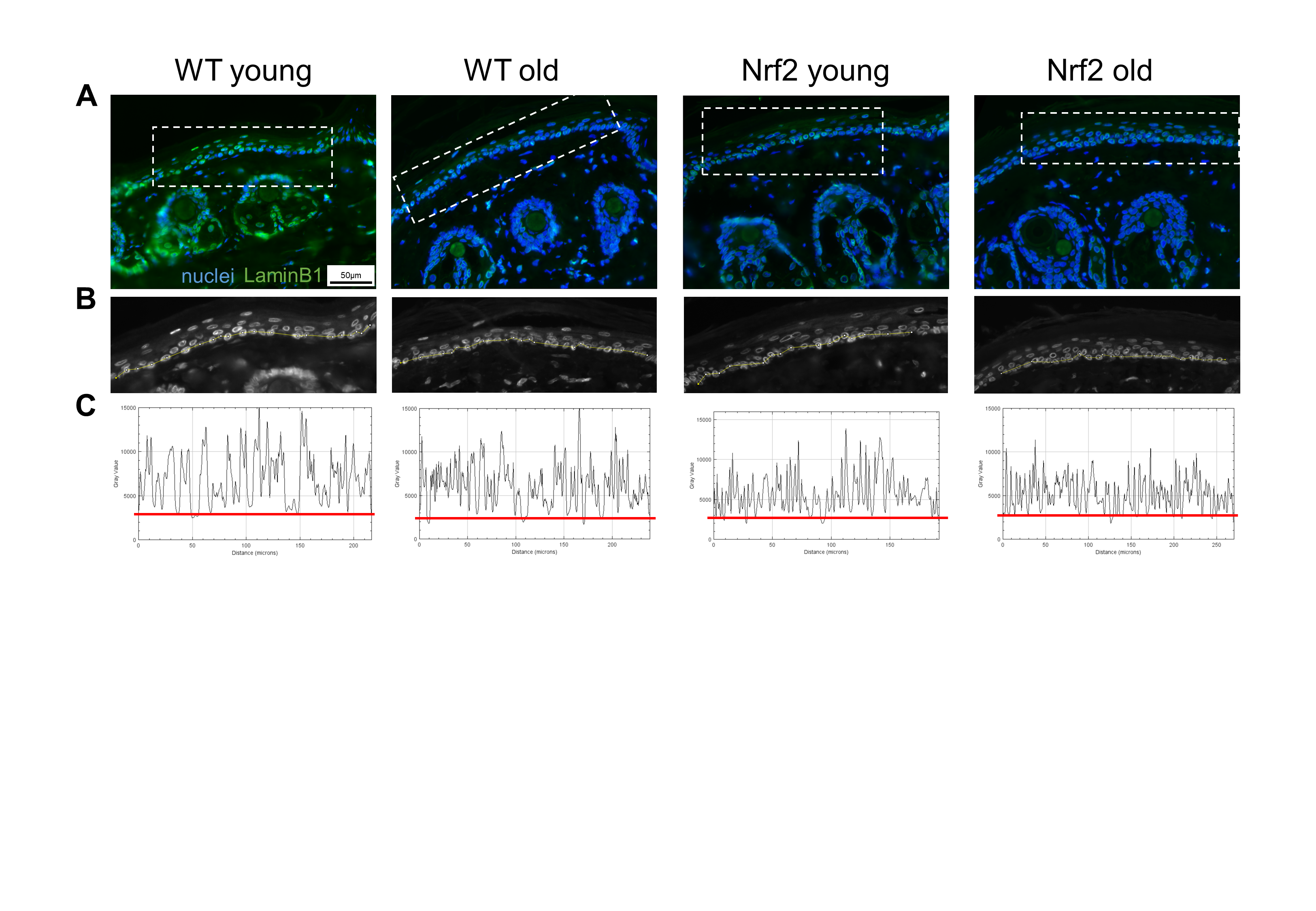

Supplement: Supplementary file 1 — Fig S1. Comparison of LaminB1 immunofluorescence staining in the epidermis of mouse tail sections (A) Representative micrographs of mouse FFPE tail sections stained for LaminB1 taken at 20x magnification. The white dashed boxes indicate the areas used for staining quantification. Scale bar: 50 μm (B) LaminB1 staining intensity was quantified along the yellow line demarking a cross section of the basal layer. (C) Plot of LaminB1 staining intensity for the given yellow line (B). Red horizontal line indicated the background noise that was subtracted for calculation of the average LaminB1 staining intensity per μm of basal layer. Quantification data of 4 field of view per mouse, 3 mice per genotype & age group. [file BIOF-49-684-s002.tif]

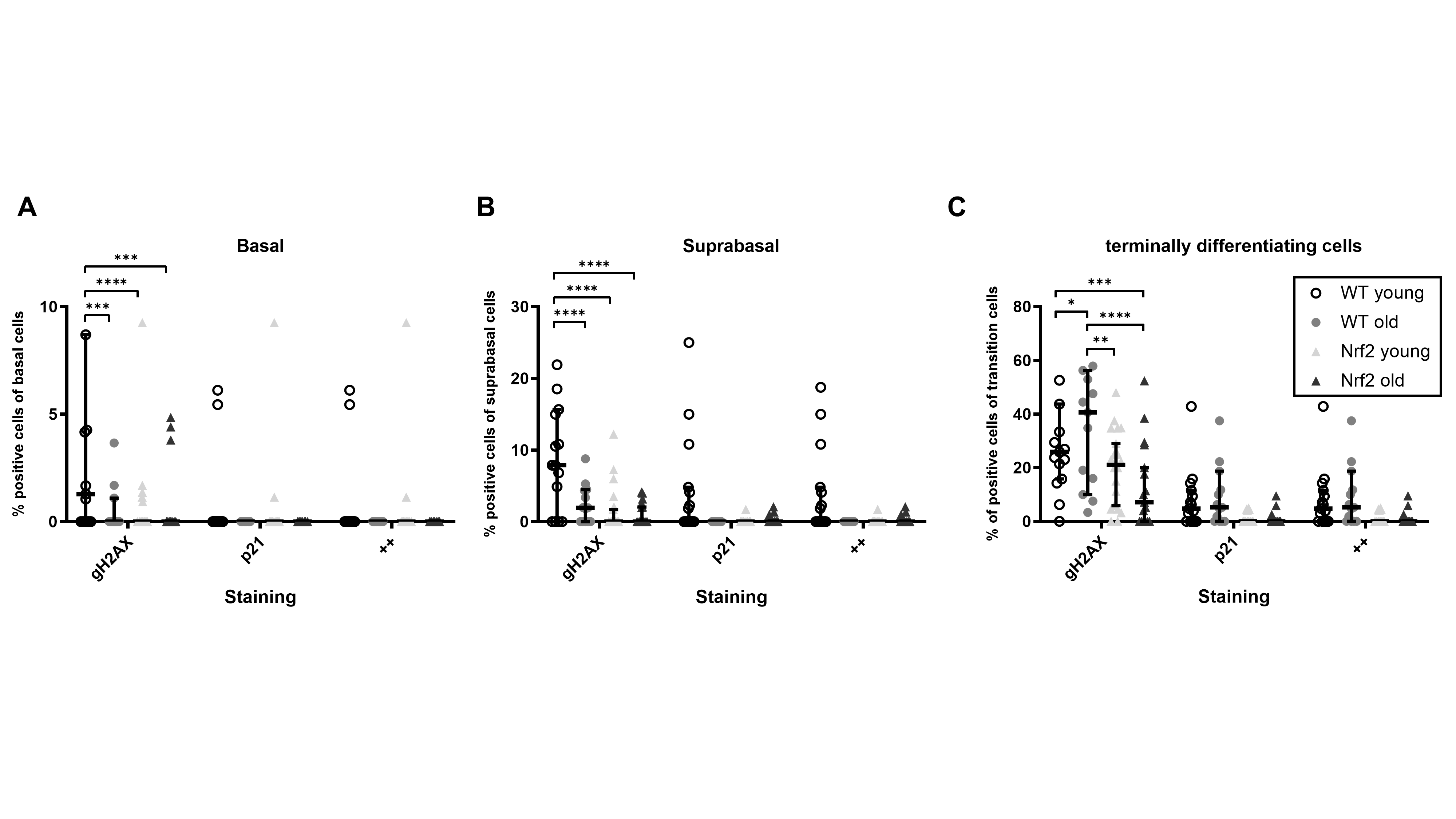

Supplement: Supplementary file 2 — Fig S2. Percentages of gamma H2AX and p21 positive cells in the different layers of the epidermis. Relative ratios of gamma H2AX (gH2AX), p21 and double positive (++) cells in the basal (A), suprabasal (B) and terminally differentiating cells (C) within the respective layer. Symbols represent quantification data from 4 field of view per mouse, 3 mice per genotype & age group. Asterisks indicate statistically significant differences (*p < 0.05, **p < 0.01, ***p < 0.001, ****p < 0.0001, one‐way ANOVA with Tukey's correction for multiple comparisons. Results are depicted as median with 95% confidence interval.) [file BIOF-49-684-s003.tif]

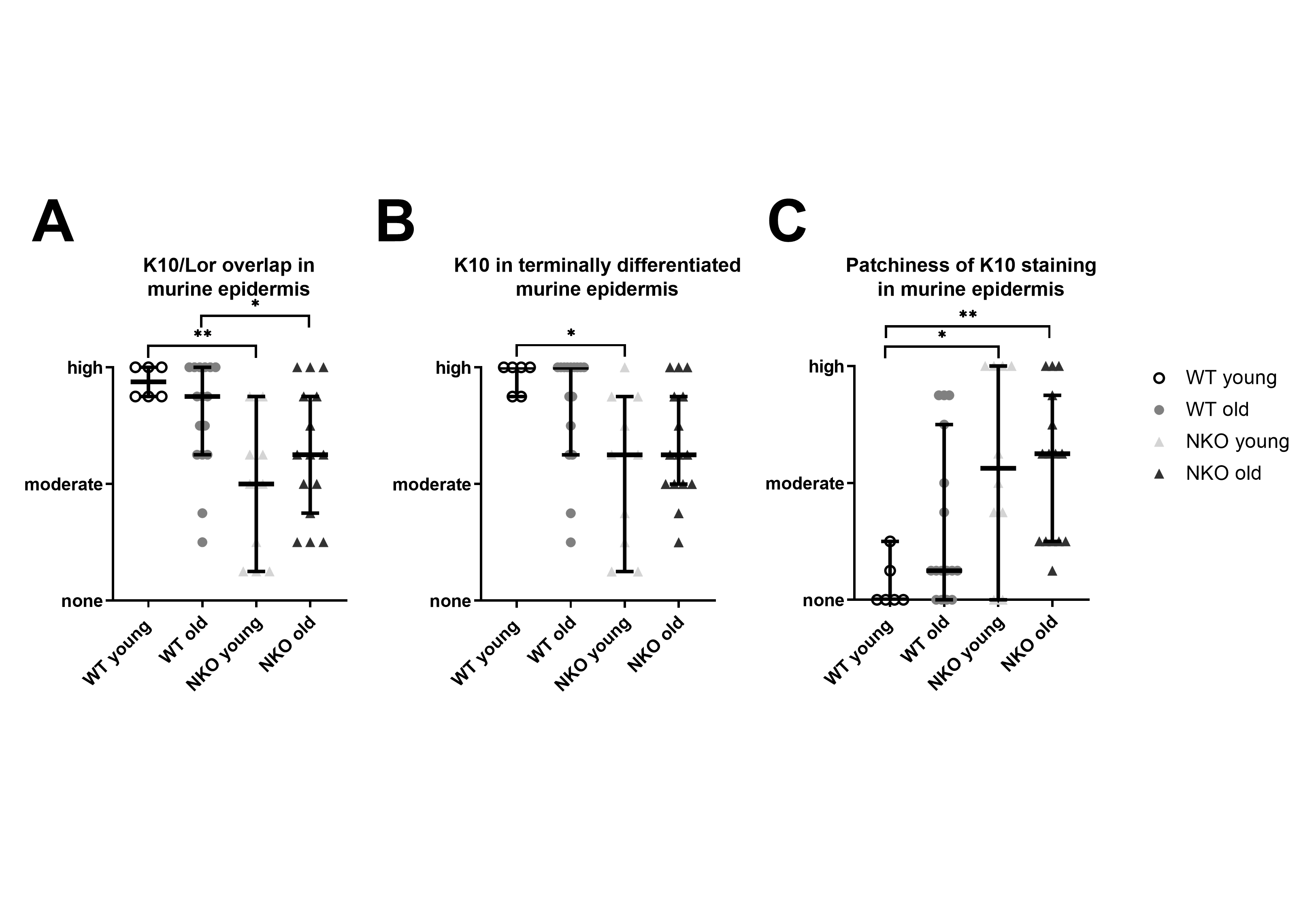

Supplement: Supplementary file 3 — Fig S3. Classification of Keratin 10 and Loricrin staining pattern in the epidermis of Nrf2 deficient murine tail cross sections of young and old wildtype (WT) and Nrf2 deficient (NKO) animals. (A) Classification of the overlap of Keratin 10 (K10) and Loricrin (Lor) immunofluorescence staining. (B) Classification of the lack of K10 from the terminally differentiating cells in murine epidermis. (C) Evaluation of the patchiness of K10 staining in the epidermis of WT and NKO mice. Classification scheme: none (not present), moderate (single cells per field of view matching the criteria), high (a large proportion of the cells per field of view match the criteria). Symbols represent the average classification score of 4 field of view per mouse, 3 mice per genotype & age group as assessed by four independent investigators. Asterisks indicate statistically significant differences (*p < 0.05, **p < 0.01, ***p < 0.001, ****p < 0.0001, one‐way ANOVA with Tukey's correction for multiple comparisons. Results are depicted as median with 95% confidence interval.) [file BIOF-49-684-s001.tif]

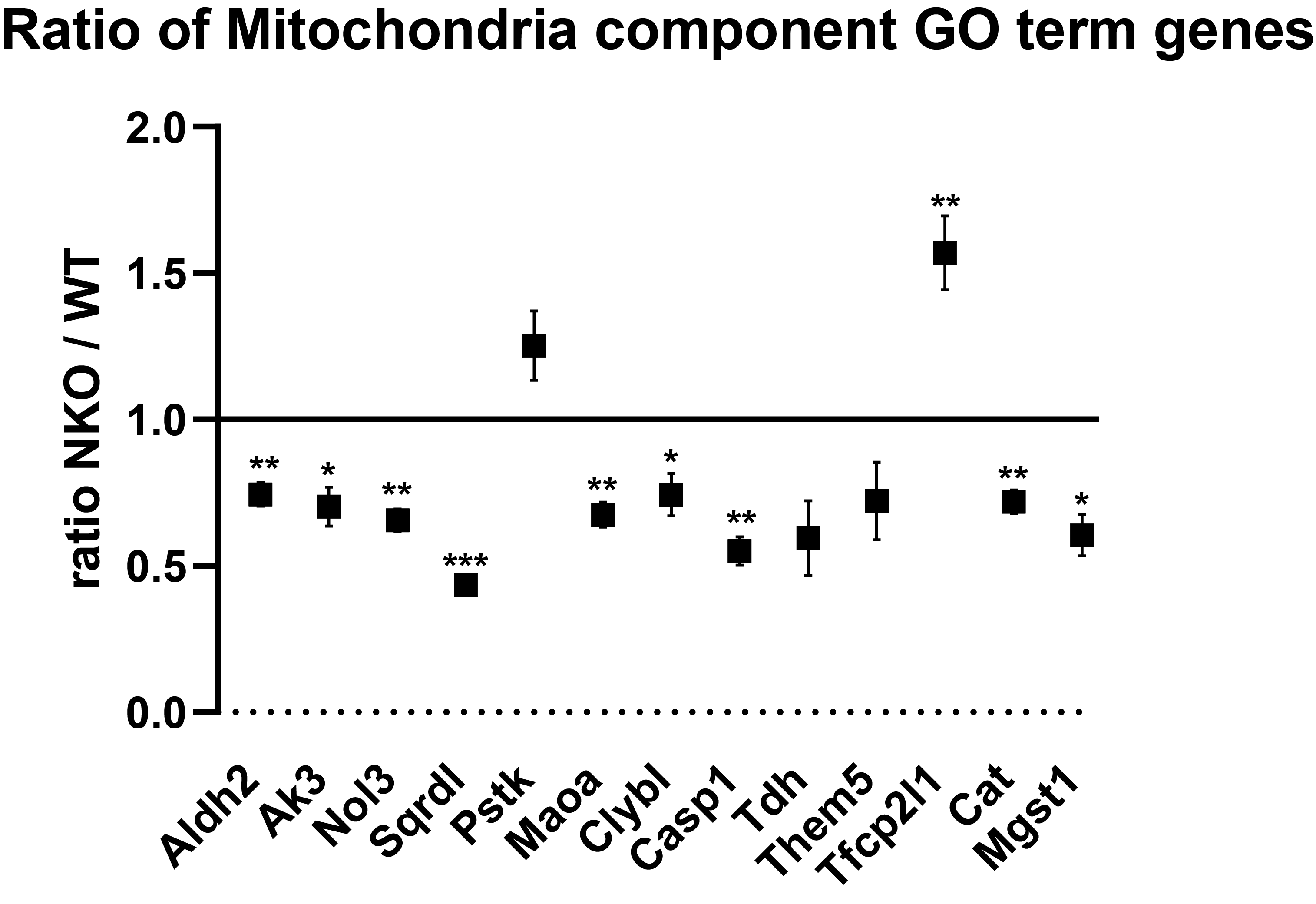

Supplement: Supplementary file 4 — Fig S4. Transcriptomic analysis of mitochondrial genes in cultured WT and NKO keratinocytes. Ratios of relative expression levels of genes tagged with the gene ontology term (cellular component: mitochondrion) from cultured WT and NKO keratinocytes. Asterisks indicate statistically significant differences (*p < 0.05, **p < 0.01, Student's t‐test. Results are depicted as mean with standard error of the mean.) Figure S5. Transcriptomic analysis of mitochondrial genes in cultured WT and NKO keratinocytes. Ratios of relative expression levels of genes tagged with the gene ontology term (molecular function ‐ chemokine OR cytokine) from cultured WT and NKO keratinocytes. Asterisks indicate statistically significant differences (*p < 0.05, **p < 0.01, Student's t‐test. Results are depicted as mean with standard error of the mean.) [file BIOF-49-684-s004.tif]
